# Efforts and systems by local governments to improve participation rates in national and local health and nutrition surveys in Japan: Findings from a workshop 2019–2024

**DOI:** 10.1371/journal.pone.0314798

**Published:** 2025-03-03

**Authors:** Midori Ishikawa, Osamu Hemmi, Yasuyo Wada, Kenichi Ohmi, Yuichi Ando, Hidemi Takimoto, Tetsuji Yokoyama

**Affiliations:** 1 Department of Health Promotion, National Institute of Public Health, Saitama, Japan; 2 National Institute of Public Health, Saitama, Japan; 3 National Institute of Health and Nutrition, National Institutes of Biomedical Innovation, Health and Nutrition, Osaka, Japan; Rikkyo University, JAPAN

## Abstract

The Japanese National Health and Nutrition Survey and local health and nutrition surveys have been used to monitor the effects of health promotion policies in Japan. However, participation rates are declining, affecting overall results. Since 2019, we have held workshops to share the efforts of local governments responsible for the survey to improve participation rates, but we have not included systems for survey implementation. Therefore, this study elucidated the efforts and systems through workshops. In 2024, 26 employees of local governments participated in the workshop using the methods developed in a previous study. The participants were divided into five groups to discuss current problems and potentially effective efforts and systems by local governments to improve participation rates. The researchers then analyzed results from the workshop, coded similar contents, and categorized similar codes and integrated them into one. These were organized into three steps (preparation for the survey, distribution and collection of the questionnaires, and following the collection of the questionnaires) for implementing surveys. The study identified that “preparation for a survey” required efforts such as “disseminating information to participants using various channels,” including “handling of the personal information of respondents.” In local structure for survey, they mentioned “cooperation with key persons such as the president of residents’ association.” For “the distribution and collection of questionnaires,” the efforts included “selecting response methods convenient for the subjects.” The systems for implementing surveys included “organization of teams composed of experienced investigators and development of members in research skills” and “specific interview practice and simulation.” For “after the collection of questionnaires,” they pointed to “holding a response standardization meeting,” and “formulating guidelines and raising awareness based on results” as efforts. From these results, the potentially effective efforts and systems for improving participation rates in the national and local health nutrition surveys conducted by local governments were elucidated.

## Introduction

National Health and Nutrition Survey in Japan (NHNS_J) was started in 1945 after World War II to assess the nutritional status and eating habits of Japanese people [1]. Since then, the Ministry of Health, Labor and Welfare (previously, Ministry of Health and Welfare) has conducted annually this survey, and the high participation rate has ensured the reliability of the results. As such, data from the NHNS_J have been used for the formulation and monitoring of national health promotion policies.

NHNS_J consists of The Nutritional Intake Status Survey, The Physical Status Questionnaire, and The Lifestyle Habits Questionnaire, administered to assess and monitor the physical condition, nutrient intake, and lifestyle of citizens based on the Health Promotion Act (Act No. 103; enacted in 2002), and to obtain basic data for the comprehensive promotion of health [2,3]. It includes surveys on nutritional intake status (home visit), physical status (with a specific venue for the physical measurements of height, body weight, abdominal circumference, and blood pressure followed by blood testing and a medical interview), and lifestyle habits (home visit or online) [2].

The field surveys of NHNS_J are conducted by public health centers established in all 47 prefectures and 105 cities or special wards with public health centers [3]. The local governments utilize the results of the NHNS_J for their health and nutrition policy planning, monitoring or conduct surveys that increased the sample size by adding areas covered by the NHNS_J. Further, some local governments may conduct their surveys, but the NHNS_J are often referred to for their analysis and results.

In recent years, however, participation rates in the NHNS_J have been declining [4,5], which is affecting the overall results [6]. Out of the three abovementioned surveys of NHNS_J, the survey on physical condition, which includes blood testing, has the lowest rate [5]. In addition, the previous study has shown the participation rate is lower for men than for women, and lower among young adults, especially those aged less than 50 years (20s–40s) compared with those aged more than 50 years [4]. Although this decline is taking place nationwide, the rate of decline varies by region [4]. The Ministry of Health, Labor and Welfare (MHLW) requests cooperation from the public [7], but increasing the cooperation rate is difficult.

In the background of this decline, it may be due to the diversification in lifestyle such as a decrease in large family households, an increase in single-person households, and an increase in the number of people living in apartment complex instead of detached houses [8,9].

The decline in participation in national surveys is likewise a major concern in several countries including Japan [10–12]; it seems related to ethnic, linguistic, or regional differences in some cases [13,14]. It should also be noted that the COVID-19 pandemic accelerated the difficulty of data collection [15]. Low or decreasing participation rate threatens the representativeness of the population in the survey data, which severely affects the generalizability of findings and the accuracy of the estimations. Approaches for enhancing the nationally representative surveys are urgently required [15,16]. Therefore, identifying strategies to increase participation rates is necessary to ensure that the data are representative of the population [17,18].

MHLW has been considering survey methods that suit the lifestyles of regions and people, and the videos related to the research methods have been released to people [19,20]. The National Institute of Health and Nutrition, which is responsible for the data tabulation of the NHNS_J, provides useful tools and information for surveys [21]. However, the participation rate continues to gradually decrease.

This declining is also an issue for local governments which utilize the survey results. Thus, public health centers responsible for conducting the survey need to consider the efforts and systems for improving participation rates.

National Institute of Public Health (NIPH) in Japan is conducting a short-term training course called “Training on techniques for monitoring and analyzing the progress of health promotion plans using health and nutrition surveys” for the personnel of local governments [22,23]. The main objective of this training is to develop the ability of local government personnel to learn survey methodologies and to acquire data analysis and utilization techniques for the health promotion plans of local governments [22].

In this training course, workshops have been held annually since 2019 to improve participation rates of the survey. Even before that, the improvement of participation rates in surveys as a need of training course had been cited from participants. We informally interviewed local government employees in charge of NHNS_J to identify their concerns and found that several local governments were “trying to improve the participation rate by being creative despite the concerns” and had “some efforts that have been successfully implemented for improving the participation rate.” However, there was no opportunity to share these efforts between local governments at that time. Therefore, we decided to conduct a workshop to enable them to share their experiences. Drafts of the methods and workshop procedures were developed by staff members drawing on their own past experiences as training staff. When we conducted the first workshop in 2019, it was well received by participants; thus, it was continued, and the methods and procedures of the workshop were improved every year considering the impressions and opinions of the participants. These results were reported in a previous study [24].

In recent years, however, COVID-19 led to the forced cancellation of multiple surveys, including the NHNS_J and other health and nutrition surveys [25], such that the need to consider the survey system in addition to efforts for improving participation rates is needed. We conducted workshops to share efforts by local governments to improve participation rates, but did not focus on systems for survey implementation. Therefore, this study identified effective efforts and systems implemented by local governments to improve participation rates in national and local health and nutrition surveys in Japan.

## Methods

### 1. Subjects and procedure

The subjects were health personnel, including registered dietitians and public health nurses, who worked in local governments and participated in the training course at the NIPH. First, the objectives and curriculum of the training were presented on the NIPH website, and a training course pamphlet was mailed to all eligible local governments (47 prefectures, and 105 cities or special wards with public health centers).

The pamphlet announced that the training would be held for four days in February 2024, and a workshop on the participation rate of NHNS_J was included on the second day [22]. After the public call for the applications from November 1 to 30, 2023, twenty-five local governments expressed interest in and registered to attend the training. We then sent a request letter that included the objective of this study and a semi-structured questionnaire to the headquarters of each local government. All 26 personnel from 25 local governments agreed to cooperate, and the researchers obtained written informed consent from all participants. This means that all participants in the training course participated in the workshop.

The items of the semi-structured questionnaire were included “What are the problems (points that need improvement) that seem to be affecting participation rates in health and nutrition surveys?” and “What efforts and systems (points that are effective/ineffective) are being taken to improve participation rates in surveys?” Furthermore, the health personnel were asked about their qualifications (e.g., registered dietitian or public health nurse) and years of administrative experience.

### 2. Workshop on efforts and systems for improving participation rates in surveys

#### 2.1 Procedure of the workshop

Table 1 showed the process in which the workshop of this study was conducted. First, the first author conducted a lecture on the issue of participation rates in the NHNS_J [3–5,10], including the results of previous workshops [24] and the objective of the current workshop (30 min). Afterward, the training staffs served as facilitators and held a workshop (120 min) on efforts and systems for improving participation rates in surveys.

**Table 1:** Workshop procedure.

		Content	Time
Lecture	0	Before the workshop, the question of participation rate in Japan’s National Health and Nutrition Survey was presented in lecture format, and the results of past workshops and the purpose of the present workshop were explained.	30 min.
Workshop	1	Drawing on the semi-structured questionnaire that the health personnel completed before the beginning of the training, each person completed a worksheet providing their opinions on the following two topics: (1) what problems seemed to decline the participation rate and (2) the efforts and systems that could be used to improve the participation rate.	20 min.
	2	The participants carried out groupwork based on the two abovementioned topics. Five groups were set up for this purpose. Each member of the group presented and shared their answers with the rest of the group and discussed the problem of participation rates. They also shared and identified the possible efforts and systems in their discussions.	20 min.
	3	The items produced by the participants were placed in an appropriate place in a 2 × 2 format (what is going well/not going well × what can be controlled/cannot be controlled). All items having the same meaning as others were treated as one item. For this, the participants were instructed not to dispute others’ opinions, to accept all opinions, and to allow all members participate.	20 min.
	4	All contents were sorted out within the group. This process was focused on things that were going well and things that could be controlled, and they were coded similar contents, categorized them, gave them names, and organized them.	20 min.
Sharing of groupwork		The controllable efforts and systems to improve the survey participation rate’ were compiled within the group and presented to other groups to exchange opinions.	40 min.

The details are as follows. A total of 26 persons were divided into five groups (one group is composed of five to six people). From the perspective of the abovementioned objective and based on the responses to the semi-structured questionnaire, each participant wrote down the current problems (points that need improvement) and the efforts and systems had implemented in their local governments (points that are effective/ineffective) on sticky notes. They then shared them with group members.

At this time, the first author explained and laid out the following rules to ensure that everyone understood and shared the information correctly: (i) write down one issue (event) per sentence, (ii) clearly indicate the subject, (ⅲ) provide specific details, (iv) point out problems and efforts and systems as your understanding, and (v) write as clearly as possible to ensure that others will understand.

Table 2 shows the matrix format of the topics addressed. The items written by each participant were placed in the appropriate place in a 2 × 2 format (points that are effective/ineffective × points that can/cannot be controlled) using this format. Items with the exact same meaning as others were considered one item. The principles that underlie this session were to acknowledge the opinions expressed by others, to accept all opinions, and to encourage the participation of all members.

**Table 2:** Matrix format of the topics addressed.

	Can be controlled^†^	Cannot be controlled ^†^
Problems (points that need improvement)	Respondents receive little reward for their participation ^‡^	No community leader in the area can persuade residents to cooperate with the survey ^‡^
Efforts and systems (points that are effective/ineffective)	Investigation assigning key persons familiar with the area.^‡^	Obtaining sufficient budget for venue usage fees ^‡^

† The problem/effort or system can/cannot be controlled by the governmental agency.

‡ Example sentences.

All contents were then organized within the group, which were focused on points that were effective and points that can be controlled. Similar contents were coded, categorized, assigned names, and organized.

#### 2.2 Results of previous workshops

In previous workshop [24], survey staff from public health centers demonstrated how they elicit survey participation using creative approaches in response to the state of residents in the target areas and households, such as family structure of household or working situation. Moreover, the workshops highlighted the importance of standardizing the survey methods because it would ensure the skills of investigators, methods for eliciting survey participation, and rewards/incentives to improve participation rates [24].

#### 2.3 Study periods

The workshop was held on February 6, 2024 in the course “Training on techniques for monitoring and analyzing the progress of health promotion plans using health and nutrition surveys.”

#### 2.4 Consensus processes in the working groups during the workshop

Each person wrote their efforts and systems on a sticky note, and two people had written the exact same thing in only rare cases. However, since there were some similar contents, each person explained their idea in more detail, providing the background of what they had written on the sticky note to the other members of the group. Then, the efforts and systems were divided into those that were expected to be effective in increasing participation rates and those that were unlikely to be effective, based on local experiences from previous surveys, by the discussion of the participants in the group. Next, drawing on all of this information, focusing on items that were expected to be effective in increasing participation rates, all of the sticky notes that were judged to be similar contents were grouped together from which categories were made, and titles were given to each category. This organization of each system into category and titled category was repeated several times until everyone was in agreement [24].

#### 2.5 Ethical considerations

This study was conducted in accordance with the guidelines laid down in the Declaration of Helsinki, and all procedures involving research study participants were approved by the Ethics Committee of the National Institute of Public Health, Wako, Saitama, Japan (NIPH-IBRA#23023, November 30, 2023).

Prior to the workshop, the researchers informed the local governments and participants of their rights during the study as follows. (1) if they feel physical or mental pain, or if they do not wish to participate or respond, they may withdraw from the study at any time; (2) declining to participate in the training will pose no disadvantage.

However, all participants re-agreed to participate in the research before, during, and after the workshop. All collected documents and personal information were protected by a password, including the data. All information and data were kept confidential. Data were stored in a locked laboratory at the organization to which the researchers belonged, who used computer that required a password log-in to access data.

### 3. Analysis of workshop results

#### 3.1 Statistical analysis of characteristics of participants and their local governments

Based on the semi-structured questionnaire, the characteristics of the participants, including their regional block of local government, age, professional qualifications, and administrative experience were analyzed. Moreover, the demographic characteristics of the participants’ local governments were compared with local governments whose officials did not participate using the Wilcoxon rank sum test. Data on the population of local governments, including age structures, rates of births/deaths, percentage of households by family structure, and life expectancies at birth, were collected from e-Stat, the portal site of official statistics of Japan [26]. All statistical analyses were performed using SAS software, version 9.4 (SAS Institute, Inc., Cary, NC, USA). A p-value of <0.05 was considered statistically significant.

#### 3.2. Analysis of the efforts and systems for improving participation rates in national and local health and nutrition surveys regarding workshop results

After the training, three researchers firstly digitized the results of all groups and integrated the contents.

The researchers manually performed data integration using the Jiro Kawakita (KJ) method. It is a research method developed by Jiro Kawakita [27,28] to classify described data.

Specifically, it includes the following steps:

*Step 1*: Read the material produced by the five groups several times to obtain an overall sense of the data. The researchers also carefully read the codes and categories for each group.

*Step 2*: Group similar codes into categories and assign names to these groups on the basis of the information given by the health personnel of local governments. Even if the categories with a similar meaning, some of them are given different names per group. If this is the case, then the researchers adopted a category name that was the easiest to understand. Finally, the codes and categories produced by the five groups were combined into one table, and the number of groups that used each code and category was written in parentheses after each item.

*Step 3*: Formulate a consensus of the final results.

*Step 4*: Position the determined codes and categories to the three steps of the NHNS_J: I. preparation for the survey, II. distribution and collection of questionnaires, and III following the collection of questionnaires.

## Results

### 1. Characteristics of the participants and their local governments

All participants in the training (26 persons) consented to participate in the research and attended all workshop sessions. None refused to participate in the study or failed to complete the training and workshop (the participation rate: 100%).

Table 3 presents the characteristics of the participants, including regional block of local government, age of participants, professional qualification, and administrative experience. The participants were composed of health personnel: 21 from 13 prefectures and 5 from cities or special wards with public health centers. Five persons were from Hokkaido and Tohoku, nine from Kanto I and Kanto II, two from Hokuriku and Tokai, two from Kinki I and Kinki II, four from Chugoku and Shikoku, and four from Kita-Kyushu and Minami-Kyushu. The average age of the participants was 38.5 years. In terms of their backgrounds, 24 were registered dietitians, and 2 were public health nurses. The average of years of administrative experience was 11 years and 4 months.

**Table 3:** Characteristics of the participants.

Number of participants			No.†
Government agency	Classification	Prefecture	21
		Cities or special wards with public health centers	5
	Regional block*	Hokkaido and Tohoku	5
		Kanto I and Kanto II	9
		Hokuriku and Tokai	2
		Kinki I and Kinki II	2
		Chugoku and Shikoku	4
		Kita-Kyushu and Minami-Kyushu	4
Personnel	Age (years)	Mean	SD^‡^
		38.5	11.1
	Professional qualifications	Registered dietitian	24
		Public health nurse	2
	Administrative experience (years)	Mean	SD^‡^
		11.4	10.2

†Number of persons.

‡SD, standard deviation.

*47 prefectures are included in the following regional blocks.

Hokkaido: Hokkaido; Tohoku: Aomori, Iwate, Miyagi, Akita, Yamagata, and Fukushima; Kanto I: Saitama, Chiba, Tokyo, and Kanagawa; Kanto II: Ibaraki, Tochigi, Gunma, Yamanashi, and Nagano; Hokuriku: Niigata, Toyama, Ishikawa, and Fukui; Tokai: Gifu, Aichi, Mie, and Shizuoka; Kinki I: Kyoto, Osaka, and Hyogo; Kinki II: Nara, Wakayama, and Shiga; Chugoku: Tottori, Shimane, Okayama, Hiroshima, and Yamaguchi; Shikoku: Tokushima, Kagawa, Ehime, and Kochi; Kita Kyushu: Fukuoka, Saga, Nagasaki, and Oita; Minami Kyusyu: Kumamoto, Miyazaki, Kagoshima, and Okinawa.

Table 4 presents the characteristics of the local governments that participated in comparison with those local governments that did not participate. Almost no difference was seen between the two groups in terms of population, age structure of the population, number of births/deaths, percentage of households by family structure, or life expectancy at birth.

**Table 4:** Comparison of demographic characteristics between municipalities that sent participants and those that did not.

			Prefectures					Cities or special wards with public health centers				
			Participated		Did not participate			Participated		Did not participate		
			n = 20		n = 27			n = 5		n = 100		
	Item		mean	SD	mean	SD	*P*	mean	SD	mean	SD	*P*
Population	Population (person)†		3154461.8	3364066.7	2335439.4	2295739.8	0.228	868308.0	544897.5	559414.6	540323.3	0.196
			median		median			median		median		
			1913771.0		1334841.0			974951.0		392683.5		
	Item		mean	SD	mean	SD	*p*	mean	SD	mean	SD	*p*
Age structure of the population (%)	Percentage of population under age 15 years †		12.3	1.3	12.1	0.9	0.906	11.7	1.8	12.2	1.2	0.615
	Percentage of population aged 15–64 years †		57.5	3.0	56.6	2.4	0.322	63.6	5.3	61.0	4.3	0.206
	Percentage of population aged 65 years or over †		30.2	3.5	31.3	2.9	0.412	24.7	4.5	26.8	4.3	0.305
Number of births/deaths (per 100,000)	Number of births (individuals)‡		648.8	102.7	624.2	59.1	0.501	678.2	82.3	686.8	100.3	0.946
	Number of deaths (individuals)‡		1233.8	182.8	1271.0	170.1	0.571	954.1	170.3	1059.5	194.5	0.201
	Number of households (households) †		42579.6	3234.1	42378.2	2326.9	0.974	48847.3	6709.2	46635.3	5142.4	0.291
Percentage of households by family structure (%)	Nuclear family households †		53.6	2.8	55.7	2.2	0.013	49.2	8.9	51.9	6.9	0.524
	Single-person households †		35.5	5.1	34.1	3.1	0.475	44.8	10.3	41.2	8.5	0.259
	Nuclear family households with a member aged 65 or older †		23.5	2.7	24.9	1.8	0.131	18.5	5.0	20.5	4.6	0.334
	Households consisting of a husband aged 65 or older and a wife aged 60 or older †		12.0	1.7	13.2	1.3	0.019	9.5	3.0	10.6	2.7	0.458
	Single-person households with a member aged 65 or older †		12.1	1.5	12.8	2.1	0.274	10.5	1.2	11.9	2.2	0.176
Life expectancy (years)	Life expectancy at birth §	male	80.6	0.7	80.7	0.5	0.915	81.2	0.2	80.9	0.8	0.284
		female	87.0	0.5	87.0	0.3	0.683	87.3	0.3	87.1	0.5	0.368

p: Wilcoxon rank sum test, SD; standard deviation.

Data source: reference 26.

†: Ministry of Internal Affairs and Communications: Population Census 2020

‡: Ministry of Health, Labour and Welfare: Vital Statistics 2021

§: Ministry of Health, Labour and Welfare: Life Tables 2020

### 2. Efforts and systems for improving participation rates in national and local health and nutrition surveys per local government

Based on the result of the analysis of the integrated data, the study identified 5 categories, 16 subcategories and 44 codes.

The categories include (1) Request for the survey participation; (2) Local structure for the survey (key person/organization); (3) Survey implementation; (4) System for survey implementation; and (5) Data input, tabulation, analysis, and utilization.

**Fig 1:**
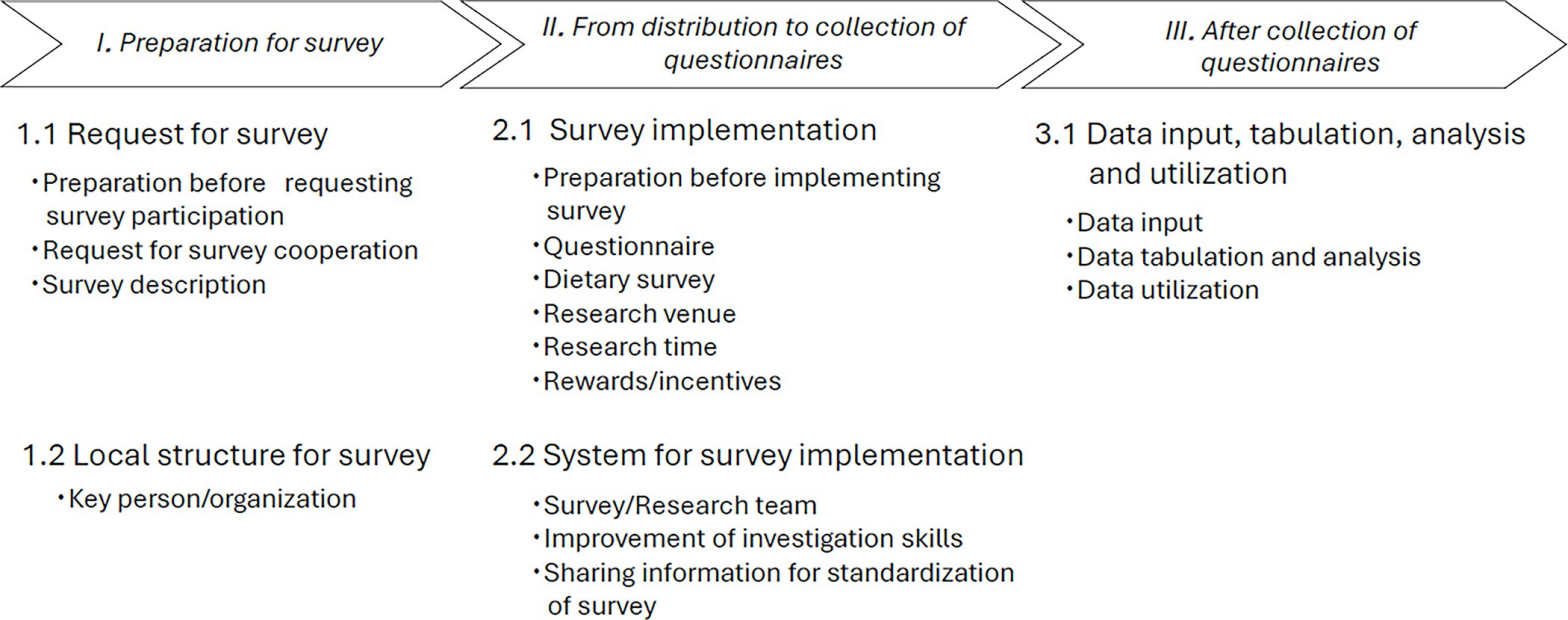
Outline of effective efforts and system implemented and potentially effective for improving survey participation rate by local governments.

Fig 1 shows an overview of the results of positioning the categories and codes in the flow of the surveys: preparation for the survey, distribution and collection of questionnaires, and following the collection of the questionnaires.

Table 5 presents the detailed contents of the efforts and systems for improving participation rates in national and local health and nutrition surveys by local governments. The numbers in parentheses indicate the number of groups and people who identified similar efforts and systems along with the specific details.

**Table 5:** Efforts and systems for improving participation rates in national and local health and nutrition surveys as per local government.

Survey flow	Category	Subcategory	Code		Example sentence
I. Preparation for a survey	1.1 Request for the survey	Preparation before requesting survey participation	Dissemination using circular boards and bulletin boards (2, 4)	–	Information about the survey is presented on the circulation board within the neighborhood association.
				–	A request flyer is posted on the bulletin board of apartment complexes.
			Dissemination using various channels such as media (3, 4)	–	Information about the survey is communicated in advance multiple times using various channels.
				–	Call for cooperation in the survey using administrative broadcasting.
				–	Promote the survey on TV and YouTube and through celebrities.
		Request for survey cooperation	Briefing sessions at a time convenient for the subjects (3, 5)	–	Hold multiple survey briefing sessions at different times.
			Consider requests based on the situation of the person being investigated (3, 3)	–	Place a reminder letter in mailbox the day before the scheduled survey date.
				–	For households that are unavailable at the time of the visit, letters and materials are placed in an envelope and dropped into the mailbox.
				–	Include contact information in the case that the subject is absent in the request letter (e.g., indicate the address of the public health center to be contacted).
			Request again to non-cooperating households (3, 4)	–	Repost the request for cooperation in the survey during the research period (e.g., send a postcard before the deadline).
				–	Visit the target household frequently at different times.
				–	Visit households that were absent from the briefing sessions together with the “district officer for the promotion of improvement in dietary habits.”
		Survey description	Explanation of the handling of personal information (3, 5)	–	Ensure that behavior and appearance are not suspicious (carry an ID).
				–	Add an official seal to the request letter and use it in a formal format.
				–	Inform all staff to be careful while handling personal information.
			Setting the survey date with the assumption of a later visit (1, 1)	–	After the briefing session, a survey date is set with a consideration of individual visits.
			Easy-to-understand explanation of materials (2, 3)	–	Provide concise explanations using easy-to-understand materials during the visit.
				–	Respond politely to questions.
			Creation of specific explanatory materials (3, 8)	–	Create materials for explanation (face-to-face).
				–	Create an explanatory video of the survey (explanations can be made non-face-to-face).
				–	Explain how to answer in an easy-to-understand manner.
				–	Create image materials (samples) of the results to be returned to individuals.
			Explanation to reduce the burden on respondents (2, 3)	–	Inform that neighboring households are also subjects.
				–	Use an online response system to indicate whether or not they would like to participate in the survey.
			Distribute supplies for the survey when requesting (1, 1)	–	Provide survey supplies (e.g., scales, measuring spoons; however, they are not rewards) in advance.
			Potential reduce part of the survey for households that refuse to participate (only in unavoidable cases; 3, 3)	–	Request to cooperate only with the lifestyle survey to households that refuse to cooperate.
				–	Inform the subjects that they may decline the blood draw (a part of the physical condition survey).
Survey flow	Category	Subcategory	Code		Example sentence
I. Preparation for survey	1.2 Local structure for survey (key person/organization)	Key person/organization	President of the residents’ association† (5, 11)	–	Explain the survey to the president of the residents’ association and obtain their cooperation in conducting the survey.
				–	Request the residents’ association about the situation of the target households.
				–	If the chairpersons of residents’ association are cooperative, then they make suggestions and take actions that lead to increased participation rates.
				–	Request the president of the residents’ association to call for residents to participate in the survey on the day of the survey.
			Administrative ward mayor (4, 7)	–	Provide an explanation of the survey to the ward mayor.
				–	Request the target households to participate in the survey by collaborating with the ward mayor.
				–	Request information to the ward mayor about the residents’ lifestyles in the survey area.
				–	Invite the target households to participate in briefing sessions by posting information on a circular board from the ward mayor.
			Municipality (4, 8)	–	Request cooperation to registered dietitians and public health nurses in municipalities.
				–	Consider and decide on a venue together with municipality officials.
				–	Request the municipal officials about the residents’ lifestyles in the survey area.
				–	Request to become a member of the survey committee and work together from the plan to evaluation.
			Key persons in area activities (3, 3)	–	Request cooperation from community-based dietitians/nutritionists.
				–	Request cooperation from the president of the social welfare council of the district.
				–	Identify key persons in districts and obtain their support.
			Prefectural Dietitian Associations (1, 1)	–	Request that prefectural Dietitian Associations request dietitian members to cooperate to be the survey investigators.
			Methods of disseminating information about the survey to areas without resident associations (1, 1)	–	Consider methods of disseminating information on the survey to areas without residents’ associations.
II. From distribution to collection of questionnaires	2.1 Survey implementation	Preparation before implementing survey	Preparing cell phones and computers for surveys (2, 4)	–	Prepare cell phones and computers for surveys.
				–	Communicate with the investigators and staff in charge via email if necessary.
				–	If the participants are unable to participate in the survey, ask them to contact the public health center in advance.
		Questionnaire	Creating an easy to answer questionnaire (2, 4)	–	Measure the survey response time and decide on the question items and numbers.
				–	The questionnaire should be expressed using easy-to-understand terms and arranged in an order that is easy to answer.
				–	In addition to the survey form, blank sheets to write down meals should be created and distributed as drafts.
			Setting response methods according to the situation of the subjects (4, 8)	–	Surveys are conducted face-to-face or online or by mail or online according to the situation of the subjects.
				–	For households that are not present, the questionnaire is placed in a mailbox and returned using a return envelope.
				–	Return envelopes also are distributed even if the face-to-face surveys (return by mail will be possible).
			Distribution of return envelopes (2, 4)	–	Create an envelope design that gives an image of the survey.
				-	– Add a list of submissions (checklist) to the return envelope to make it easier to understand.
Survey flow	Category	Sub-category	Code		Example sentence
II. Distribution and collection of questionnaires	2.1 Survey implementation	Dietary survey	Addition of supplementary information in the sheet of dietary records by investigators (2, 3)	–	Ask the participants about their meal contents and add supplementary information in the sheet for dietary records.
			Request to provide photos of meals (3, 5)	–	Request to take and provide photos of actual food intake.
				–	Present food labels, packaging, and photos to confirm the contents of meals.
			Request to provide food packages (2, 2)	–	Request to provide packages of purchased foods shown in the dietary records.
		Research Venue	Setting venues according to the convenience of subjects (3, 9)	–	Select a venue familiar to participants.
				–	Select a venue (e.g., community center and meeting place) as close as possible to the surveyed households.
				–	The venue for the Physical Status Questionnaire could be a place next to a school club.
				–	Utilize places familiar to the child-rearing generation (e.g., children’s plaza) as the survey venue.
			Secure a budget for venue usage fees (1, 1)	–	Secure a budget for venue usage fees.
			Secure a venue with a comfortable room temperature (1, 1)	–	Ensure a venue with a comfortable room temperature.
			Gather information and decide on venues together with municipal officials and ward mayors (2, 2)	–	Determine the venue after confirming with municipality officials or ward mayors.
			Cooperation with venue managers/persons in charge (2, 2)	–	Obtain the cooperation of persons in charge at the venue.
				–	Explain the survey to the representative/manager of the venue and gain their understanding.
		Research time	Setting and disseminating the survey time according to the convenience of the subjects (4, 9)	–	Set up a time for implementing the survey, such that participants can come after work.
				–	Inform the times when there is less waiting time for the survey at the venue.
				–	Conduct surveys on weekends (Saturday and Sunday) and at night.
		Rewards/incentives	Explanation and distribution of rewards at the time of sending the request for a survey and briefing sessions (2, 5)	–	Give rewards when requesting a survey or briefing session.
				–	Explain in advance the content of the reward that will be given after the investigation is completed.
			Attractive rewards/incentive (4, 5)	–	Daily life necessities (e.g., tissue paper, garbage bags designated by municipalities).
				–	Gift certificate.
				–	Facility usage ticket.
				–	Grant points that are beneficial to consumers.
				–	Return of individual dietary survey results.
			Rewards that match the burdened placed by the survey on respondents (1, 1)	–	Identify whether or not the reward is commensurate with the burden of surveys.
Survey flow	Category	Subcategory	Code		Example sentence
II. Distribution and collection of questionnaires	2.2 System for survey implementation	Survey/research team	Team organization centered on experienced investigators (3, 6)	–	Organize teams around experienced investigators and ensure that skills and knowledge are transferred to new investigators and personnel in charge.
				–	Appoint a person who is familiar with the area/community as an investigator.
				–	Ensure that investigation teams assign key persons familiar with the area.
			Training of new investigators and personnel in charge (3, 4)	–	Assign a new dietitian/nutritionist to the venue of the Physical Status Questionnaire.
				–	Pair experienced and inexperienced investigators to transfer the skills of experienced investigators and train the next generation.
				–	Conduct onsite training for new investigators.
			Efforts to secure investigators (3, 5)	–	Request the prefectural Dietitian Association to recommend an investigator for survey.
				–	Consider students as investigators as part of onsite training at registered dietitian training institutions and universities and educate them.
				–	Take budgetary measures to increase the number of days in which investigators are employed.
				–	Work with a freelance nutritionist.
		Improvement in investigation skills	Training sessions and seminars to improve investigation skills (3, 6)	–	Conduct training sessions for investigator candidates.
				–	Hold training sessions for the staff in charge of surveys and standardize techniques and methods.
				–	Hold training sessions for collaborators of the survey (community/ home-based dietitians/nutritionists) to improve their skills on a regular basis.
				–	Hold a briefing session based on the case study for the staff in charge.
			Specific interview practice and simulation (2, 4)	–	Practice interviews through role playing.
				–	Conduct a simulation before visits.
		Share information for the standardization of surveys	Sharing of information to avoid discrepancies between public health centers (2, 3)	–	Exchange information (between the dietitians of public health centers) regarding community/home-based dietitians/nutritionists.
				–	Close communication and share questions and answers for survey to prevent differences in understanding between public health centers.
				–	Use the written records of experienced investigators about meal contents based on interviews as a sample for other investigators to fill out.
			Information sharing between investigators and staff in charge (1, 3)	–	Quickly share information between the investigator and staff in charge via email, chat, and phone, etc.
III. After the collection of questionnaires	3.1 Data input, tabulation, analysis, and utilization	Data input	Data input (1, 3)	–	Hire a person to aggregate data through the prefectural Dietitian Association and input data efficiently.
				–	Hold a meeting for those in charge and standardize the answers.
				–	Create a list of replacement data for answer contents that are not standard and build the database.
		Data tabulation and analysis	Data tabulation and analysis (1, 2)	–	Outsource aggregation and analysis of data.
				–	Speed up the publication of results by outsourcing the tabulation analysis of data.
		Data utilization	Data utilization (1, 1)	–	Develop dietary guidelines based on the survey results for enlightenment.

Numbers in brackets represent the numbers of groups and participants that made effort or system.

Numbers in brackets represent the numbers of groups and members that provide such effort or system.

† President of the residents’ association pertains to the representative or responsible person of the residents’ association in the area and organizes local events such as summer festivals, new year events and disaster drills.

Numbers in brackets represent the numbers of groups and participants that make effort or system.

#### 2.1 Preparation for the survey

*2*.*1*.*1* For preparation for the survey, the respondents identified “dissemination using circular boards and bulletin boards” and “dissemination using various channels such as media” as efforts that fall under “preparation before requesting survey participation”.

It was pointed out the need for “briefing sessions at a time convenient for the subjects” and “repeating requests to non-cooperating households” as efforts for “request for survey cooperation”.

In terms of survey description, they discussed “explanation of the handling of personal information” and “easy-to-understand explanation of materials”.

*2*.*1*.*2* For “local structure for the survey (key person/organization),” the consensus was on “explain the survey to the president of the residents’ association and administrative ward mayor, and obtain their cooperation in conducting the survey,” and “request municipal officials to become a member of the survey committee and work together from the plan to evaluation”.

#### 2.2 Distribution and collection of questionnaires

*2*.*2*.*1* For “survey implementation,” which is under preparation before implementing survey, the respondents suggested “preparing cell phones and computers for surveys” and “creating an easy-to-answer questionnaire”.

For the dietary survey, they emphasized the “addition of supplementary information in the sheet of dietary records by investigators” and “request to the provision of photos of meals”.

Local governments have also exerted considerable efforts regarding the venue and time of the survey, including “setting venues according to the convenience of subjects” and “setting and disseminating the survey time according to the convenience of the subjects”.

For rewards and incentives, they suggested that rewards should be explained and distributed at the time of the request for a survey and briefing sessions.

In addition, they shared that reward items that are attractive to participants, such as daily necessities (e.g., tissue paper and garbage bags), gift certificates, and the return of the individual results of the dietary survey, are effective.

*2*.*2*.*2* In terms of systems for survey implementation, they highlighted the importance of the survey team and mentioned “team organization centered on experienced investigators” and “ensure that skills and knowledge are transferred to new investigators and personnel in charge.” To improve investigation skills, they proposed “specific interview practice and simulation” in addition to “sharing information to avoid discrepancies between public health centers”.

#### 2.3 Following the collection of questionnaires

For data input, tabulation, analysis, and utilization, they suggested to “hire a person to tabulate data through the prefectural Dietitian Association and input data efficiently” and “speed up the publication of results by outsourcing the tabulation and analysis of data”.

## Discussion

This study identified the systems implemented (and those that had the potential effectiveness) by local governments seeking to improve participation rates in national and local health and nutrition surveys. Previous workshops elucidated helpful aspects from efforts to increase participation rates but did not focus on systems for survey implementation [24]. The present study has therefore systematically focused on efforts and systems.

Furthermore, the study identified five categories during the flow of the survey as follows: (1) Request for survey participation; (2) Local structure for survey (key person/organization); (3) Survey implementation; (4) System for survey implementation; and (5) Data input, tabulation, analysis, and utilization.

We observed many exchanges of experiences and opinions on the importance of preparing for surveys, how to request a survey, and local structure (key persons and organizations). In this way, we considered what kind of initiatives would be effective. For example, we identified effective efforts that informed the target area and households with circular boards, bulletin boards, and multiple channels before requesting cooperation in the survey; afterward, briefing sessions could be held at convenient times for the subjects.

As a system for promoting these efforts, one of the respondents showed that the key to improving participation rates was to provide detailed explanations to key persons and organizations in the area and obtain their cooperation in encouraging participation from residents. Thus, it was thought that it would be necessary to consider methods of disseminating information with respect to the survey in areas without a residents’ association.

The National Health and Nutrition Examination Survey in the United States also suggests that conducting visits for requests and interviews for the survey is important for improving participation rate. It described that getting people to understand about the survey during visitations might be related to the decrease in the number of people who subsequently drop out of the surveys [11]. Furthermore, for the 2021–2022 survey, the guiding principles changed to ensure the safety of the survey participants and field staff from the COVID-19 pandemic [29].

In the Danish National Health Survey, a report on participation rates emphasized the importance of increasing the rate of cooperation in surveys [30]. The study pointed out that the cover page of a survey, including the request text, might be as important as the survey and that the content, language, format, and layout might influence nonparticipation [14].

In addition, for effective efforts, the current study raised some points on the importance of the creation of explanatory materials for use during visits to the households of the subjects, such that they can obtain a concrete image of the content of the survey. In doing so, they recommended that the specific details of the incentives for participation should be clearly explained to the subjects such as returning individual results and indicating the details of the reward. MHLW is already working on related initiatives [19,20], but it is necessary to pursue further strengthening of these efforts.

Several previous studies demonstrated that incentives and rewards are related to participation rates in surveys [10,31,32]. They were indicated that providing a reward prior to a survey is significantly effective, which is in line with the results of the current study. Another study suggested that in younger generations, this strategy is more effective for men than for women [33].

Furthermore, the study presents a novel finding, in conducting surveys, the effective structure for implementing the survey is important, along with the specific efforts. In specifically, opinions were also exchanged about forming teams centered on veteran/experienced investigators and devising methods for transferring their skills and knowledge to new investigators from them. They indicated that undergoing training is important for local government staff and investigators, including practical training sessions with role playing and simulation exercises, and that exchanging information is necessary to avoid discrepancies between public health centers within local governments.

This study has several limitations. First, only 26 individuals from 25 local governments participated out of the 157 local governments in Japan. Variations existed in regional characteristics and years of administrative experience among group members. These differences may have influenced individual remarks and group dynamics. However, the results of efforts in this study were almost similar to those observed in workshops conducted from 2019 to 2023 in which 59 local governments participated. In addition, there was hardly any difference between the local governments whose officials were participants and those without participants in terms of population, age structure of population, number of births and deaths, percentage of households by family structure, or life expectancy at birth. Moreover, the current results of the system or structure for conducting surveys cannot be compared with previous ones, because they are a new finding.

Another potential concern is that the qualitative methodology for category formation limits the generalizability of the results to other Japanese prefectures. Nevertheless, these findings provide a basis for future quantitative investigations. Additionally, this study confirms that, similar to a previous study [24], local government officials are interested in improving participation rates and quality of responses at the same time.

The efforts and systems identified by the current study will be applied to training materials used in training sessions for local governmental personnel in charge of surveys at the NIPH. Furthermore, in the local governments, it will be used for their actions toward the improvement of participation rates in surveys.

## Conclusion

This study identified the efforts and systems which were implemented and potentially effective by local government during the process of surveys to improve participation rates in national and local health and nutrition surveys.
